# Spatial mapping of posture-dependent resistance to passive displacement of the hypertonic arm post-stroke

**DOI:** 10.1186/s12984-023-01285-7

**Published:** 2023-12-01

**Authors:** Priyanka Kanade-Mehta, Maria Bengtson, Tina Stoeckmann, John McGuire, Claude Ghez, Robert A. Scheidt

**Affiliations:** 1 Joint Department of Biomedical Engineering, Marquette University and the Medical College of Wisconsin, Engineering Hall, Rm 342, P.O. Box 1881, Milwaukee, WI 53201-1881 USA; 2 Department of Physical Therapy, Marquette University, Milwaukee, USA; 3 Department of Physical Medicine and Rehabilitation, Medical College of Wisconsin, Milwaukee, USA; 4 Department of Neuroscience, Neurology, and Physiology, Columbia University Medical Center, New York, USA

**Keywords:** Hypertonia, Passive stretch, Velocity-dependent torque response, Tonic stretch reflex, Electromyography, Elbow, Shoulder

## Abstract

**Background:**

Muscles in the post-stroke arm commonly demonstrate abnormal reflexes that result in increased position- and velocity-dependent resistance to movement. We sought to develop a reliable way to quantify mechanical consequences of abnormal neuromuscular mechanisms throughout the reachable workspace in the hemiparetic arm post-stroke.

**Methods:**

Survivors of hemiparetic stroke (HS) and neurologically intact (NI) control subjects were instructed to relax as a robotic device repositioned the hand of their hemiparetic arm between several testing locations that sampled the arm's passive range of motion. During transitions, the robot induced motions at either the shoulder or elbow joint at three speeds: very slow (6°/s), medium (30°/s), and fast (90°/s). The robot held the hand at the testing location for at least 20 s after each transition. We recorded and analyzed hand force and electromyographic activations from selected muscles spanning the shoulder and elbow joints during and after transitions.

**Results:**

Hand forces and electromyographic activations were invariantly small at all speeds and all sample times in NI control subjects but varied systematically by transport speed during and shortly after movement in the HS subjects. Velocity-dependent resistance to stretch diminished within 2 s after movement ceased in the hemiparetic arms. Hand forces and EMGs changed very little from 2 s after the movement ended onward, exhibiting dependence on limb posture but no systematic dependence on movement speed or direction. Although each HS subject displayed a unique field of hand forces and EMG responses across the workspace after movement ceased, the magnitude of steady-state hand forces was generally greater near the outer boundaries of the workspace than in the center of the workspace for the HS group but not the NI group.

**Conclusions:**

In the HS group, electromyographic activations exhibited abnormalities consistent with stroke-related decreases in the stretch reflex thresholds. These observations were consistent across repeated testing days. We expect that the approach described here will enable future studies to elucidate stroke's impact on the interaction between the neural mechanisms mediating control of upper extremity posture and movement during goal-directed actions such as reaching and pointing with the arm and hand.

## Introduction

Muscles in the post-stroke arm commonly demonstrate abnormal reflexes that result in increased position- and velocity-dependent resistance to movement (i.e. spastic hypertonus: [21, 25]. Spastic hypertonia is understood to reflect systematic reductions in stretch reflex thresholds [16, 24], decreased range of regulation of these stretch reflex thresholds [23, 47], as well as altered non-reflex phenomena such as abnormalities in the intrinsic mechanical properties of spastic muscles and altered viscoelastic properties of passive tissues [28, 43, 49]. Importantly, systematic reduction in stretch reflex threshold could lead to significant increase in stretch reflex excitability [17] and agonist/antagonist coactivation in some regions of the workspace [22, 30], which could lead to complex, posture-dependent and potentially time-varying joint impedances in the hemiparetic arm. Because the feedforward control of goal-directed movements relies on accurate predictions of limb impedance [38], spatial and temporal complexity of joint impedance may be a significant contributor to impairment of movement coordination post-stroke [20, 40, 48]. Although a velocity-dependent increase in muscle tone (spasticity is frequently assessed clinically and has been quantified in single joints such as the elbow and wrist [15, 23, 26, 44], quantitative assessments of multi-joint control deficits have been rare (see [30, 36]) and sometimes rely on techniques such as kinematic and/or inverse dynamics analyses, which can be insensitive to the presence and effects of abnormal muscle coactivations (e.g., [18, 33]).

The goal of this study was to develop a reliable approach for measuring the mechanical consequences of abnormal neuromuscular mechanisms as a function of hand location in the reachable workspace in the hemiparetic arm post-stroke. We are motivated in this work by a growing body of experimental evidence supporting the idea that the neural mechanisms contributing to the control of limb posture and movement can be differentially compromised by stroke [19, 27, 40], see also [8, 37, 48] and by other neuromotor disorders (cf. [10, 11]), and by the belief that greater understanding of the mechanisms contributing to sensorimotor deficits will eventually lead to improved efficacy of therapeutic interventions [13]. Our work builds on prior studies that examined transient mechanical and electromyographic responses to passive displacements of the wrist or elbow to quantitatively assess post-stroke spasticity (cf. [15, 23, 26, 44, 47]) and spastic dystonia [46]. In one example, Schmit and colleagues used a motorized device to passively flex and extend the elbow of hemiparetic stroke survivors over a range of speeds ranging from slow (6º/s) to fast (90º/s) [44]. Their goal was to assess the reliability of three different biomechanical correlates of spasticity, which they isolated from other aspects of spastic hypertonia associated with dystonia, contracture, and increased joint stiffness. They did so by subtracting the torque response to the slowest displacement from responses to faster displacements, leaving only reflex torque. This approach is effective because the stiffness of the passive tissues about the elbow joint are largely velocity insensitive [9]. Of the three biomechanical measures considered—peak torque, peak joint stiffness, and onset angle of reflex torque responses—peak torque values measured during displacements at 90°/s were most reliable on repeated measures in a single testing session (> 80% reliability), and most highly correlated with clinical assessments of spasticity (Ashworth Scale). In another example, Mirbhageri and colleagues [29] used system identification techniques to quantify the contributions of reflex and intrinsic (i.e., non-reflex) stiffness to total elbow stiffness at several different elbow angles in the paretic and nonparetic arm of chronic hemiparetic stroke survivors. Each position was examined under passive conditions in the range of full elbow flexion to full elbow extension. They reported that intrinsic and reflex stiffness both contributed strongly to net joint torque, that the effects were significantly larger in the paretic than in the non-paretic elbow muscles, and that these differences increased with the increasing joint angle indicating position dependence. Although these prior studies suggest that manifestations of spasticity and hypertonia may vary in complex ways across the reachable workspace after stroke, a more comprehensive approach to quantifying mechanical expressions of spasticity and hypertonia across the workspace has yet to be described.

We designed a set of experiments using a two-joint, planar robot to measure the dynamic and quasistatic mechanical and electromyographic responses to controlled displacements of the upper extremity at several locations in the arm’s workspace. Subjects were instructed to relax as the robot moved their hand sequentially between target locations spanning the reachable workspace at speeds ranging from very slow to fast. Trajectories were selected such that movements were largely limited to either the shoulder or elbow joint, but not both. The robot stabilized the hand at the target for at least 20 s following the end of each movement. We analyzed the time series of horizontal planar hand forces and electromyographic (EMG) activations for selected arm muscles to determine the duration of phasic, velocity-dependent resistance to stretch, to characterize the spatial topography of tonic, position-dependent hand forces throughout the workspace, and to characterize the muscle activations that give rise to these postural bias forces. The resulting data demonstrate that the robotic assessment of posture-dependent bias forces was repeatable across days, that the phasic component of these stroke-related forces lasted no more than 2 s after the end of limb re-positioning, and that these bias forces were partly neuromuscular in origin (not merely due to passive tissue resistance to stretch) such that elevated “resting” EMG activations exhibited posture-dependence in some muscles, but posture-invariance in others. We expect that quantitative evaluation of posture-dependent bias forces may facilitate future assessments of stroke's impact on the interaction between the control of upper extremity posture and movement.

## Methods

**Fig. 1:**
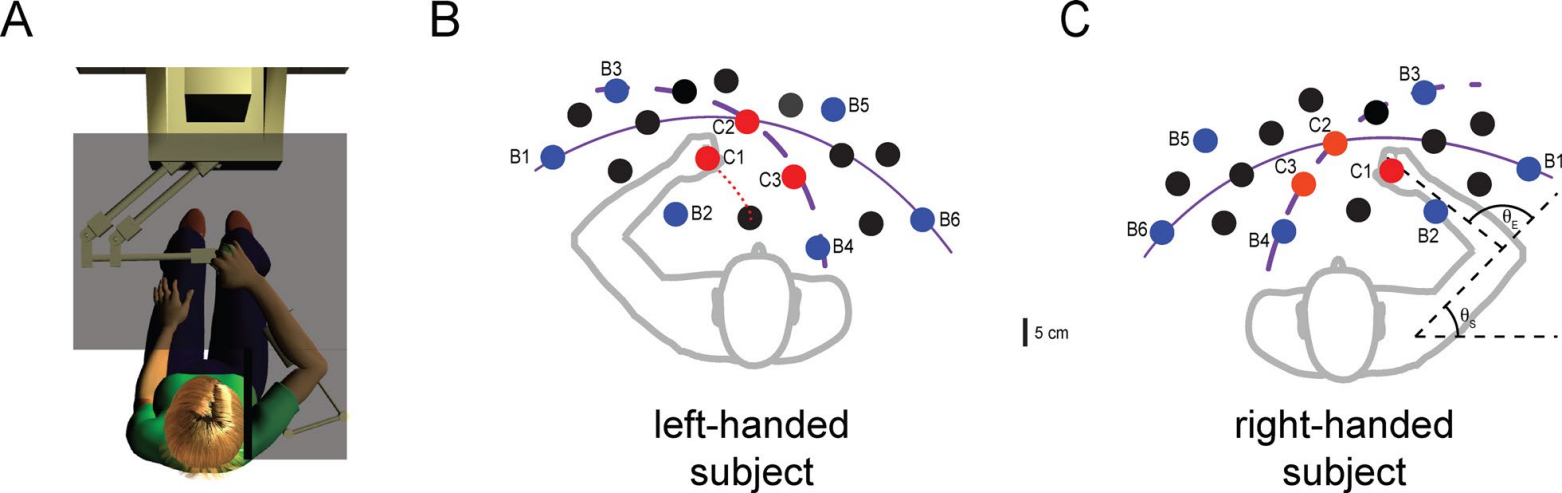
Experimental setup. **A** Subject seated at the horizontal planar robotic testing system. The system included the robotic tool as well as a horizontal planar feedback display and electromyography sensors (not shown). **B** Representative left-hand workspace with sampling points. The robot enforced transitions between targets along paths designed such that motion of the subject’s arm occurred predominantly at one joint (either the shoulder or elbow) while the other joint remained essentially still. Dashed and solid purple arcs: exemplar elbow and shoulder trajectories, respectively. Detailed analyses were performed at selected sample locations labeled as boundary (B1 through B6, blue circles) or center points (C1 through C3, red circles). Interior points are shown in black. Red dotted line: a transition analyzed in greater detail below (Fig. 3). **C** Representative right-hand workspace with sampling points. Note that the left- and right-arm workspaces and joint angle definitions (θ_S_ and θ_E_) were mirror-symmetric such that the hand was at boundary point B4 when the shoulder and elbow both were flexed to the maximum extent allowed by the subject’s passive range of motion and/or the robot’s workspace

A convenience sample of ten unilateral, hemiparetic survivors of stroke (HS; aged 43–62 years) and nine neurologically intact control subjects (NI; age-range matched: 43–61 years) gave informed consent to participate in this study (see Appendix 1). All procedures were approved by Marquette University’s Office of Research Compliance in accord with the Declaration of Helsinki. All HS were recruited from the pool of hemiparetic stroke outpatients of Medical College of Wisconsin and the Milwaukee VA Medical Center and all were in the chronic stage of recovery (between 2- and 28-years post-stroke). Exclusion criteria for HS included: inability to give informed consent, inability to follow 2-step directions, history of tendon transfer in the affected limb, physical dimensions prohibiting appropriate interaction with the robotic assessment system (Fig. 1) despite reasonable adjustment attempts (e.g. abdominal intrusion into the robot’s workspace or an inability to see the feedback display after chair height adjustment), use of aminoglycoside antibiotics, curare-like agents, or other agents that might interfere with neuromuscular transmission, botulinum toxin treatment within the previous 8 months, and/or shoulder pain in the test position of 75 to 90° abduction. These exclusion criteria were designed to ensure participant safety and to eliminate confounding factors that could compromise the measurement and interpretation of abnormal hand forces arising from abnormal neuromuscular responses to imposed hand displacements. The presence of contracture or shoulder subluxation did not exclude subjects from participating unless it limited their ability to perform the experiments comfortably. NI control subjects were required to have no history of neurological disorder. All subjects were able to achieve all test positions without discomfort. Three of the NI subjects participated in three experimental sessions with at least one week separating each session. The remaining six NI subjects participated in a single session. Seven HS participated in two experimental sessions separated in time by at least one week. The remaining HS participated in three experimental sessions typically separated by at least one week. Each session lasted ~ 2 h.

*Clinical assessments*—Prior to experimentation, HS participated in a series of clinical assessments. The upper extremity portion of the Fugl-Meyer assessment of motor performance (FM_UE_) was used to assess impairment in the ability of each HS to move the more affected arm [7]. Scoring for the FM_UE_ is on a 0–66 point scale, which is based on the subject’s ability to move both within and outside of muscle synergies. Increasing values reflect greater motor control. The Modified Ashworth Scale (MAS) was used to assess hypertonia in the flexors and extensors at the shoulder, elbow, and wrist. Scoring for the MAS is on a 0–4-point scale with 0 indicating no increase in tone and a 4 indicating that the more-affected limb is rigid in flexion or extension. Within each limb, MAS scores were averaged across the three tested joints to obtain an overall estimate of upper extremity spasticity (range: 0–4; see also [48]). Passive range of motion of the elbow and shoulder in the workspace plane was measured using a goniometer both with and without the robotic test apparatus. Handedness was assessed for all subjects using the Edinburgh Handedness Inventory [31]. HS were asked to use pre-stroke preferences to guide their answers. Appendix 1 provides a description of subject characteristics for all subjects. Within the group of survivors, FM_UE_ scores ranged from 19 to 53, reflecting a broad spectrum of functional motor impairment within our subject pool.

*Experimental procedures*—Subjects were seated in an adjustable high-backed chair fixed in front of an actuated, 2-joint robotic manipulator designed to move in a horizontal plane (see [39]). Subjects were strapped into the chair using a seatbelt-style chest harness that minimized trunk motion. Efforts were taken to situate subjects in the same location and orientation relative to the robot from one testing day to the next. An opaque screen mounted 1 cm above the plane of hand motion occluded vision of the arm, hand, and robot arm. The subject’s wrist was splinted at 0° flexion and fixed at the wrist to the robot’s handle with Velcro straps. The arm was supported against gravity (between 75 and 90° abduction angle) using a lightweight, chair-mounted support. Arm segment lengths and passive range of motion at the shoulder and elbow were used to determine each individual’s reachable workspace in the horizontal plane. A minimum of 18 spatial sample locations (targets) spanned the full range of shoulder and elbow flexion and extension (Fig. 1B and C) except in subjects with reachable workspaces exceeding the robot’s workspace. In those cases, the sample locations spanned the intersection of the subject's range of motion and that of the planar robot. For each subject, detailed data analyses were performed at three targets defined as center locations (red dots labeled C1–C3 in Fig. 1B and C) and at six targets defined as boundary locations (blue dots labeled B1–B6 in Fig. 1B and C). The remaining points were used to construct spatial maps of posture-dependent interface forces and muscle activations as described below. Subjects were verbally coached throughout the experiment to “relax” at all times and not to intervene as the robot generated passive movements of the arm through its workspace. To reinforce our verbal coaching, the word RELAX was projected onto the occluding screen throughout the experimental session.

The robot monitored hand position using rotational encoders (A25SB17P180C06E1CN; Gurley Instruments Inc., Troy, NY) mounted on the motor shafts (M-605-A Goldline; Kollmorgen, Inc. Northampton, MA). The location of the handle could be resolved within 0.038 mm throughout the operating workspace. A 16-bit data acquisition board (PCI-6031E DAQ; National Instruments Inc., Austin, TX) sampled analog force data from a load cell (85M35A-I40-A-200N12; JR3 Inc., Woodland, CA) that was mounted immediately under the handle. The robot was programmed to generate stiff control of hand position; data collection and robot control were performed using the Matlab XPC toolbox (Mathworks, Inc., Natick, MA) at a rate of 1000 samples per second (see also [14]).

The robot enforced transitions between targets along paths designed so that motion occurred predominantly at one joint (the focal joint: either the shoulder or elbow) while the non-focal joint remained essentially still. Figure 1B and C show elbow and shoulder trajectories (dashed and solid purple arcs, respectively) along which only the focal joint was flexed or extended. Desired hand paths were computed in real-time using a previously published approach [34] based on anthropometric measurements of upper and forearm limb segment lengths and localization of the participant’s shoulder relative to the origin of the robot’s workspace. Hand trajectories had bell-shaped speed profiles computed such that rotation of the focal joint had peak angular velocities that were slow (6°/s), medium (30°/s), or fast (90°/s). In contrast to step- or ramp-shaped velocity profiles, bell-shaped profiles achieve smoother transitions between one location and another, imitating the smooth changes in arm position applied during manual testing of muscle resistance by clinicians [24]. After the end of transition (EOT), the robot held the hand at the target location for at least 20 s (the holding period). Targets C1–C3 were visited from all four directions (elbow extension, elbow flexion, shoulder extension, and shoulder flexion) in pseudorandom order. Targets at a workspace boundary (B1-B6) were visited from at least two directions in pseudorandom order. In total, 105 transitions were used to visit all target from all desired directions at the three different speeds (also pseudorandomized across trials).

*Data Collection and Analysis*—Our experimental setup allowed us to synchronously record instantaneous hand position and force as well as surface electromyograms (EMG) from: shoulder horizontal adductors pectoralis major (PECS) and anterior deltoid (ADL), shoulder horizontal abductor posterior deltoid (PDL), elbow flexors biceps (short and long heads: BICS and BICL) and brachioradialis (BRD), as well as elbow extensors triceps (lateral and long heads: TRILT and TRILG). Raw EMGs were amplified × 1000 (Myosystem 1200, Noraxon, Inc. Scottsdale, AZ) and band-pass filtered between 10 and 500 Hz in hardware prior to digitization. 60 Hz and 120 Hz artifacts were eliminated in post-processing using zero-phase, 4th-order Butterworth notch filters. Residual offsets were removed from the digitized EMGs prior to rectification and low-pass filtering at 4 Hz with a zero-phase, 4th-order Butterworth filter. All data (kinematics, forces, and EMGs) were streamed to disk during the experiments for subsequent off-line processing.

To facilitate comparison of EMGs across the study population, each subject’s muscle activations were normalized by the peak value of the rectified and filtered activation recorded from that muscle during a series of 5 s duration maximum voluntary isometric contractions (MVICs). MVICs were performed with the hand stabilized in the center of the arm’s horizontal planar workspace using a rigid handle fixed to the robot frame. Each subject performed 12 MVIC trials before the experiment: 3 each of maximal isometric elbow flexion, elbow extension, shoulder horizontal abduction and adduction (cf. [38, 45]). Peak EMG activation for each muscle was defined as the largest EMG value for that muscle within any MVIC trial after signal processing as described in the preceding paragraph. We also recorded 5 s of quiet resting activation in the same limb configuration prior to MVIC recording after approximately 5 min of rest, wherein subjects were encouraged to “relax”.

A primary goal of our analysis was to differentiate velocity-dependent resistance to stretch post-stroke from position-dependent, steady-state effects. To this end, we filtered the kinematic and kinetic time series off-line using a zero lag, 4th order, 10 Hz Butterworth low-pass filter and analyzed the hand’s force vector at nine points in time including the moment of peak hand speed (V_MAX_), the moment of target acquisition (End of Transition or EOT, defined as the time when hand velocity first dropped below 15% of its peak value), as well as at 1, 2, 3, 5, 10, 15, and 20 s after EOT. We also analyzed the magnitude of rectified and filtered EMG signals at the same time points.

*Statistical testing—*We performed two main analyses. The first analyzed interface forces at the robot's handle to quantify velocity-dependent resistance to passive motion of the arm. We controlled for posture-dependencies to determine the earliest point in time after EOT beyond which hand force ceases to demonstrate velocity-dependence. This was done to better isolate posture-dependent effects in the second analysis described below. As we will show, hand forces measured at the end of the holding period displayed no systematic dependence on movement speed, and so we regarded hand forces measured at EOT + 20 s as representative of asymptotic values. We subtracted these values from hand forces measured at other time points in the same trial before performing a six-way, repeated measures, general linear model ANOVA examining how changes in measured hand forces (relative to EOT + 20 s) varied by subject group (HS or NI), movement speed (6°/s, 30°/s, 90°/s), sampling instant (time of peak transit velocity, EOT, and 1, 2, 3, 5, and 15 s after EOT), movement direction (elbow flexion, elbow extension, shoulder flexion, shoulder extension), workspace location (center vs. boundary, wherein the center locations were collapsed across C1–C3 and the boundary locations were collapsed across B1-B6) and testing day (1–3). Post-hoc ANOVA and Tukey t-tests were performed to examine significant main and interaction effects.

The second analysis characterized the spatial topography of tonic, position-dependent hand forces as a function of workspace location at the earliest time point wherein hand forces neither depended on transport velocity nor differed from the asymptotic values. To do so, we plotted raw hand force vectors as a function of workspace location for each subject. These plots were then co-registered with contemporaneous maps of normalized EMG activations to determine whether the observed hand forces might be partly neuromuscular in origin. Specifically, we evaluated the extent to which shoulder and elbow muscle activations varied as a function of joint angle along the solid (PECS, ADL, and PDL) and dashed lines (BICS, BICL, BRD, TRILT, and TRILG) in Fig. 1B and C.

Data processing and statistical testing were carried out within the Matlab (Mathworks, Inc., Natick, MA) and Minitab (Minitab Inc., State College, PA) computing environments. Effects were considered statistically significant at the α = 0.05 level. Specifically, we applied a Bonferroni correction to the 20 statistical tests performed on the hand force data, which yielded an individual test significance threshold of p = 0.0025. Despite consistency in the mechanical recordings, the EMG data demonstrated considerable variation across stroke survivors. Consequently, we did not correct the EMG analyses for multiple comparisons (i.e. we accepted statistical significance at p = 0.05).

## Results

**Fig. 2:**
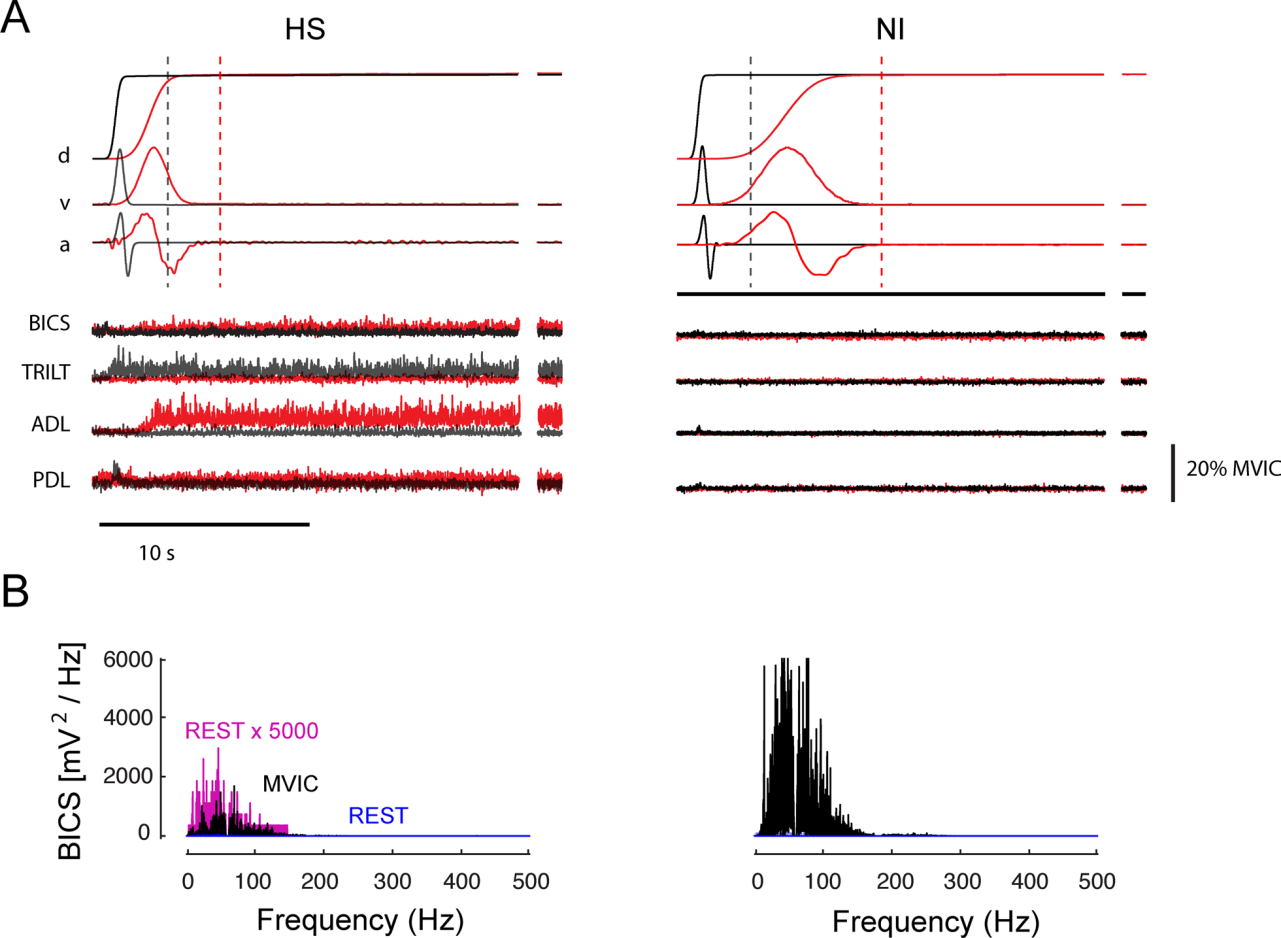
**A** Representative normalized movement kinematics (top) and normalized, rectified EMG signals (bottom) as a function of time for a single fast transition between targets C1 and B4 (grey) and a single moderate speed transition between targets C2 and B1 (red) in a representative HS subject (left). A single fast transition between targets C1 and B4 (grey) and a slow transition between targets C2 and B1 (red) in a representative NI control subject is shown on the right. Horizontal scale bar: 10 s. Vertical scale bar: EMG signal amplitude of 20% MVIC. Movement kinematics are displayed on an arbitrary scale to highlight general characteristics such as smoothness. The additional snippets of data to the right of each trace present data from the very end of the hold period (at least 25 s after the end of movement). Vertical dashed lines indicate the time of EOT + 2 s. **B** Spectrograms of BICS EMG signal power during MVIC trials (black) and quiet rest (blue) for the same subjects. Also shown for comparison for the HS subject (left) is the resting spectrogram multiplied by a factor of 5000 (purple)

All subjects were alert throughout each experimental session even though they were not asked to engage in any task-dependent activation aside from relaxing. Robot-generated hand movements were always smooth, having unimodal, bell-shaped velocity profiles (Fig. 2A). Passive displacement of the hand modulated EMG activation in HS subjects (especially in stretched muscles) but rarely did so in NI control subjects. For example, stretching shoulder or elbow muscles in the hemiparetic arm caused EMG activation in some muscles to increase during movement (Fig. 2A, left), as might be expected due to spastic hypertonia (i.e. [41]. Interestingly, elevated muscle activation could remain active long after motion had ceased in some regions of the workspace, as might be expected due to a decrease in the stretch reflex threshold post-stroke [23], although other explanations are possible. In some muscles, tonic EMG activation after passive displacement of the hand appeared to depend on the final limb configuration (limb posture). Persistence of muscle activation was never observed in NI control subjects during or after passive movement (cf. Figure 2A, right).

Elevated EMG activations in HS subjects were not an artifact due to poor signal transduction or improper EMG normalization in these subjects. Because EMG normalization with respect to MVIC could overestimate voluntary muscular effort if the signals were corrupted by environmental noise, we visually verified that EMGs recorded from each muscle were of high quality by plotting the EMG signal power spectrograms from MVIC trials for each muscle and compared it to spectrograms obtained during quiet rest. In all cases, MVIC EMG signal power was distributed across the frequency range in a unimodal pattern characteristic of high-quality surface EMG recordings [35] (Fig. 2B, black). By contrast, EMG signal power was lower during rest in the center of the workspace (Fig. 2B, blue). MVIC EMG exceeded quiet resting signal power by a factor greater than 100 in all cases (≫ 20 dB signal to noise ratio). Thus, normalization with respect to MVIC yielded a high-quality assessment of relative voluntary effort. Note that while resting EMG power was uniformly negligible at all frequencies in NI subjects, it commonly displayed measurable, tonic activation post-stroke with a spectral signature of quality surface EMGs rather than broadband environmental noise (e.g. Figure 2B, left; purple).

**Fig. 3:**
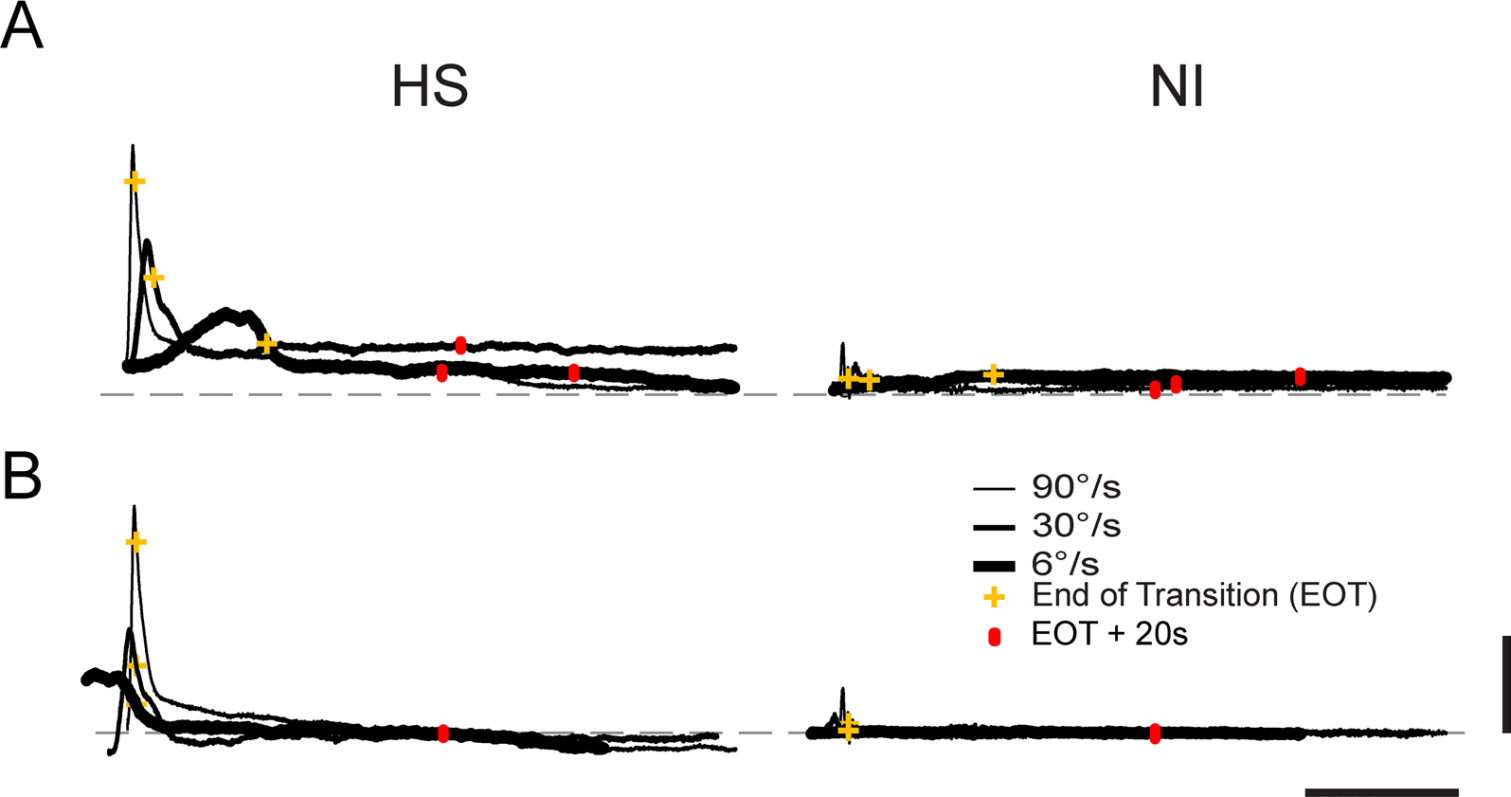
**A** Hand force magnitudes as a function of time during transitions between targets as indicated by the red trajectory in Fig. 1 (panel **B**) for a representative HS subject (left) and NI control subject (right) at three transport speeds (90˚: thin trace; 30˚: medium-weight trace; 6˚: heavy trace). Markers (yellow +) indicate the time of End Of Transition (EOT). Markers (red hashes) indicate EOT + 20 s. Vertical scale bar corresponds to a measured hand force of 20N while the horizontal dashed line indicates 0 measured hand force. The horizontal scale corresponds to 10 s. **B** Force profiles plotted with respect to steady-state values (EOT + 20 s) and aligned in time with respect to EOT. Here, the vertical scale bar corresponds to a change in measured hand force of 20N *relative to steady-state*

We sought to characterize how mechanical resistance to passive joint motion varied with movement velocity (i.e., spastic hypertonia; [21]. Visualization of raw hand forces from a selected HS subject (Fig. 3A) demonstrated that hand force magnitude could vary systematically by transport speed during movement, that variations in steady-state hand force were not systematic during the hold phase that followed, and that hand forces changed very little from EOT + 20 s onward. Despite efforts to minimize trunk motion, some trial-by-trial variability in hand force undoubtedly arose from subjects shifting in their seat during the ~ 1.5-h testing period. Because the effect of these infrequent postural shifts would be to alter the bias forces recorded at the handle throughout the trial, we reasoned that greater sensitivity in subsequent analyses of velocity-dependence would be achieved by removing the effects of postural shifts (i.e. by analyzing hand forces relative to their asymptotic values). Indeed, exploratory three-way repeated measures ANOVA found that hand force magnitude at EOT + 20 s post-stroke did not vary systematically with transport speed (F_(3,207)_ = 1.40, p = 0.25). We therefore aligned the raw hand force profiles in time with respect to EOT and subtracted asymptotic values on a trial-by-trial basis (Fig. 3B) prior to evaluating how subject group, workspace location and other factors influence the transient effect of movement on reaction forces at the hand.

### Hand force measurements are repeatable across days

We performed a six-way, mixed-model, general linear model, repeated measures ANOVA to quantify how changes in measured hand force magnitude (relative to EOT + 20 s) varied by subject group, movement speed, movement direction, workspace location (boundary vs center), temporal sampling instant and testing day in response to passive relocation of the subject’s hand from one workspace location to another. We found that testing day failed to demonstrate a significant main effect (F_(2,4799)_ = 0.26, p = 0.769) and that this factor also had no interaction with any other factor (p > 0.21 in each case). Moreover, 78% of the Day 2 variations in hand force (across subjects, workspace location, speed, movement direction and sampling window) were predicted by measurements made on Day 1. From this we conclude that measurement of endpoint forces using our experimental approach was repeatable across days.

### Effect of movement speed on measured hand forces

Although the ANOVA found no main effect of movement direction or workspace location on hand force (relative to steady-state) and no significant interaction between subject group and either of these factors, the analysis did find a significant three-way interaction between subject group, sampling instant and movement speed (F_(14,4799)_ = 3.02; p < 0.0005). No other three-way or higher-order interactions achieved significance, indicating that the observed velocity-dependent effects did not vary systematically across the workspace after stroke. To test whether the observed three-way interaction could possibly have been due to alterations in the passive viscoelastic properties of tissues spanning the shoulder and elbow joints post-stroke, we repeated the ANOVA on the sampled hand force data from the 30°/s and 90°/s trials after subtracting values obtained during the 6°/s trials (i.e. trials wherein velocity-dependent stretch reflex activity—but not viscoelastic resistance—should have been minimal; [41, 44]. We obtained similar results and identical statistical conclusions from this supplemental analysis (results not shown). Thus, velocity-dependent responses measured during and shortly after movement were not solely due to passive tissue viscoelasticity but rather implicated the presence of abnormal stretch reflexes post-stroke.

**Fig. 4:**
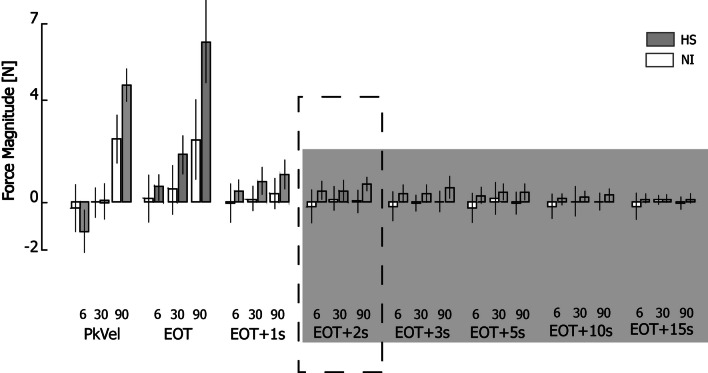
Force magnitude as a function of speed, time and subject group (HS: Shaded bars; NI: open bars). Error bars represent ± 1 SEM. The shaded area from EOT + 2 s onwards indicates the sampling intervals in which ANOVA found no effect of movement speed on hand force magnitude. The dashed box identifies the point in time selected for subsequent detailed analysis of hand force and EMG data

Figure 4 plots change in hand force magnitude relative to steady-state averaged across target locations, movement directions, and days. The three-way interaction between subject group, sampling window and movement speed can readily be seen in that hand forces differed across groups, transport speeds, and sampling times during and shortly after transport but not later in the holding period. To determine the earliest point in time beyond which hand force ceased to be movement velocity-dependent, we performed a post-hoc series of eight separate two-way, mixed-model, general linear model, repeated measures ANOVA to determine how changes in hand force magnitude varied by subject group and movement speed for each of the eight sampling times (PeakVel, EOT, and EOT plus 1, 2, 3, 5, 10 and 15 s). In contrast to the first three sampling times, wherein the main effect of movement speed was either significant (PeakVel: F_(2,38)_ = 70.58, p < 0.0005; EOT: F_(2,38)_ = 21.14, p < 0.0005) or marginally significant (EOT + 1 s: F_(2,38)_ = 2.76, p = 0.085), the main effect of movement speed was absent at EOT + 2 s (F_(2,38)_ = 0.91, p = 0.417) and at all subsequent time points. As shown by the vertical dashed lines in Fig. 2A, arm movement had completely ceased prior to EOT + 2 s (i.e. there was no acceleration or velocity component associated with EOT + 2 s for any movement speed). The interaction between movement speed and subject group was significant only during movement (PeakVel: F_(2,38)_ = 8.44, p < 0.002); no interactions were found at later times. A set of one-sided t-tests revealed that hand force magnitude post-stroke did not differ from asymptotic values from EOT + 2 s onwards. Thus, spastic responses (i.e. velocity-dependent resistance to stretch) are most visible in the post-stroke hand in the fastest conditions of limb transport, but only *during* transport or for a short period thereafter (i.e. less than 2 s after movement ceases).

### Effect of hand position on postural bias forces

**Fig. 5:**
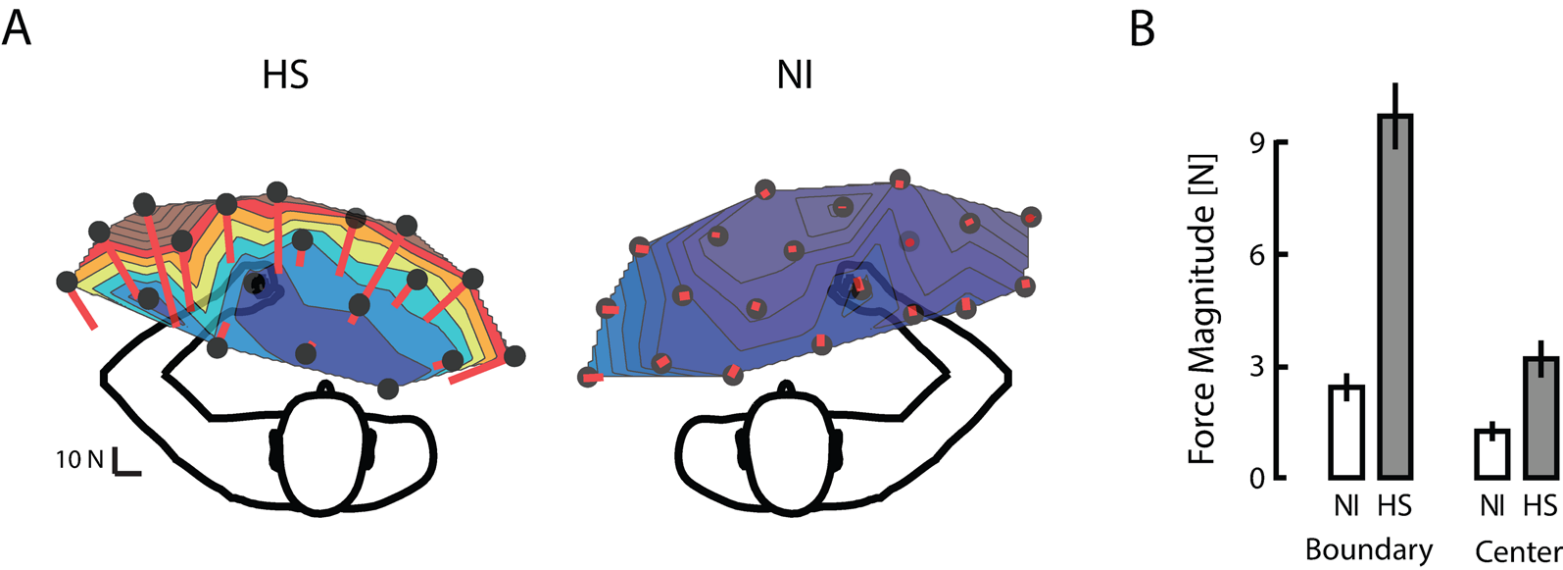
**A** Raw hand force vectors as a function of location throughout the workspace for a representative HS subject (left) and NI subject (right). **B** Population summary of hand force magnitude as a function of workspace location (boundary vs. center) for HS (grey bars) and NI subjects (open bars) at EOT + 2 s. Error bars represent ± 1 SEM

Because hand forces reached velocity-independence and steady-state by EOT + 2 s, we next analyzed the raw hand forces—not relative to asymptote—measured at EOT + 2 s for each subject. Representative data are shown in Fig. 5A, where raw hand force magnitude was averaged across movement speeds and days. Compared to NI subjects who generated negligible tonic forces at the robot's handle throughout the workspace after movement had ceased, each HS subject displayed a unique field of hand forces that varied systematically across their arm’s reachable workspace. Steady-state, posture-dependent hand forces were typically higher at the workspace boundaries in HS subjects. These forces were stronger on one side of the reachable workspace and pointed towards an equilibrium point located in the approximate center of the workspace. These observations were supported by results of a mixed-model, repeated measures ANOVA that examined how hand force magnitude values measured at EOT + 2 s varied as a function of subject group (HS, NI), movement direction (EF, EE, SF, SE), and workspace location (boundary, center). Workspace location demonstrated a strong interaction with subject group (F_(1,37)_ = 51.24, p < 0.0005): hand forces varied strongly depending on whether the location was at or near the boundary or center of the workspace for HS (Fig. 5B) but not for NI subjects. The ANOVA found no evidence supporting a main effect of movement direction or any interaction between this and the other factors (p > 0.301 in all cases). Thus, hand forces measured at EOT + 2 s post-stroke were predominantly position-dependent, having no systematic dependence on movement speed and/or direction.

### Elevated hand forces are accompanied by large EMG activations in some hemiparetic arm muscles post-stroke

**Fig. 6:**
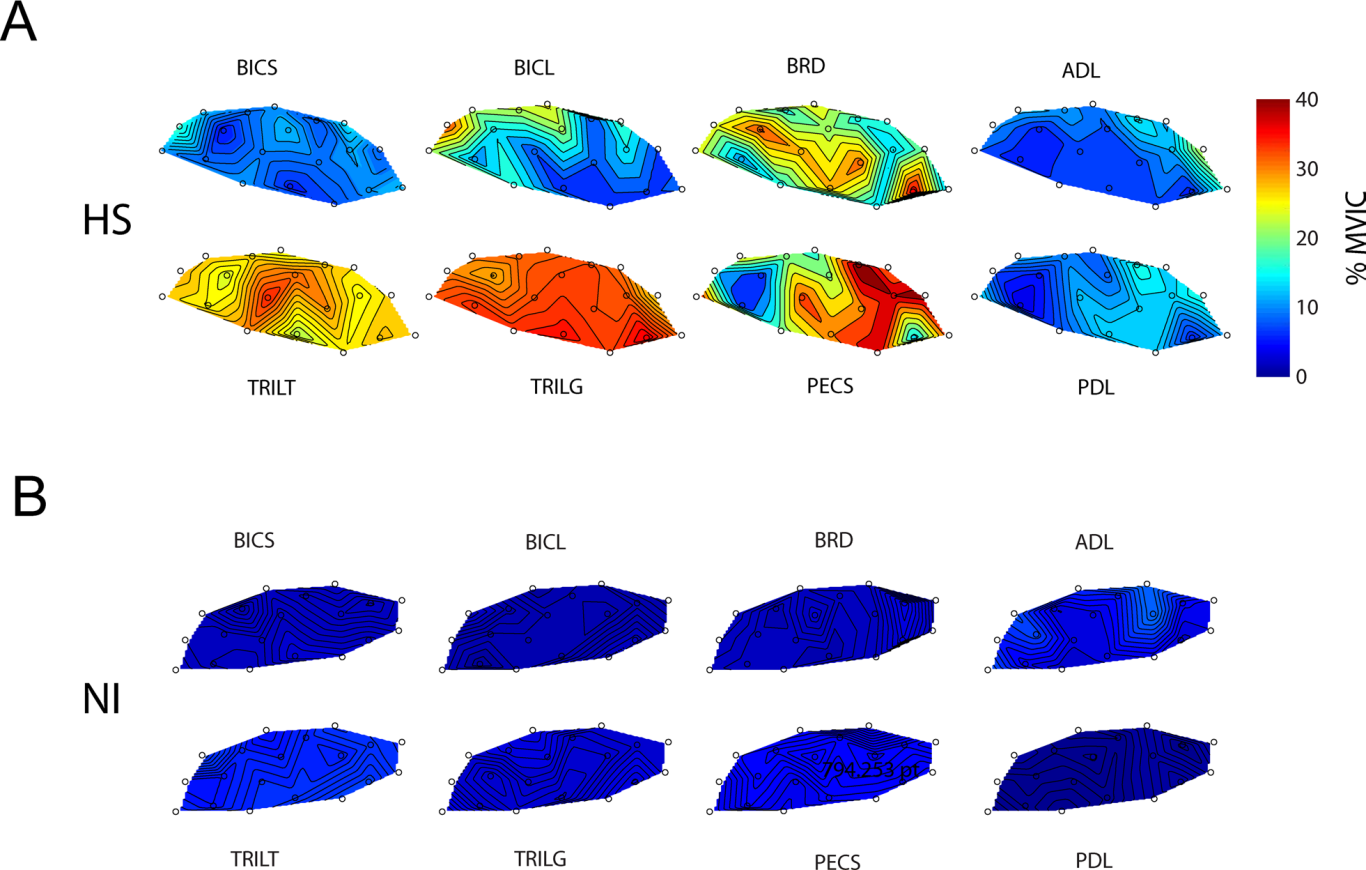
**A** Contour plots of elbow and shoulder muscle activations (averaged across transition speeds) for a selected HS subject at 2 s after End of Transition. Muscle activation is presented as a function of hand position in the workspace on a scale ranging from 0 to 100% maximum isometric voluntary contraction (color bar on the right). **B** Contour plots of elbow and shoulder muscles for a selected NI subject at 2 s after End of Transition

We next sought to determine whether posture-dependent bias forces post-stroke were due primarily to passive properties of tissues spanning the hemiparetic joints or whether bias forces were at least partly neuromuscular in origin. Figure 6 plots spatial maps of selected EMG activations (normalized to MVIC) at EOT + 2 s for a representative subject from each group. For the selected HS subject, robotic translation of the hand led to elevated levels of EMG activations that were relatively large in some muscles with respect to signals recorded during maximal voluntary isometric contractions (e.g., TRILT, TRILG and BRD). In other muscles, the activations tended to exhibit posture-dependence such that activation was greater when the muscle was lengthened than when shortened (e.g., BICL, PECS). Yet other muscles exhibited negligible activations at EOT + 2 s regardless of limb posture (e.g., BICS, ADL, PDL). These results were characteristic of the study population in the sense that all stroke survivors exhibited high levels of muscle activation throughout the workspace only in some muscles (most notably TRI and PECS), a modest tendency to exhibit posture-dependent activation in a one or two muscles (which varied by individual), and no indication of abnormal “resting” activation in the remaining muscles. By contrast, activation was minimal in all muscles by EOT + 2 s throughout the workspace for all NI subjects (a selected individual's results are shown in Fig. 6B).

**Fig. 7:**
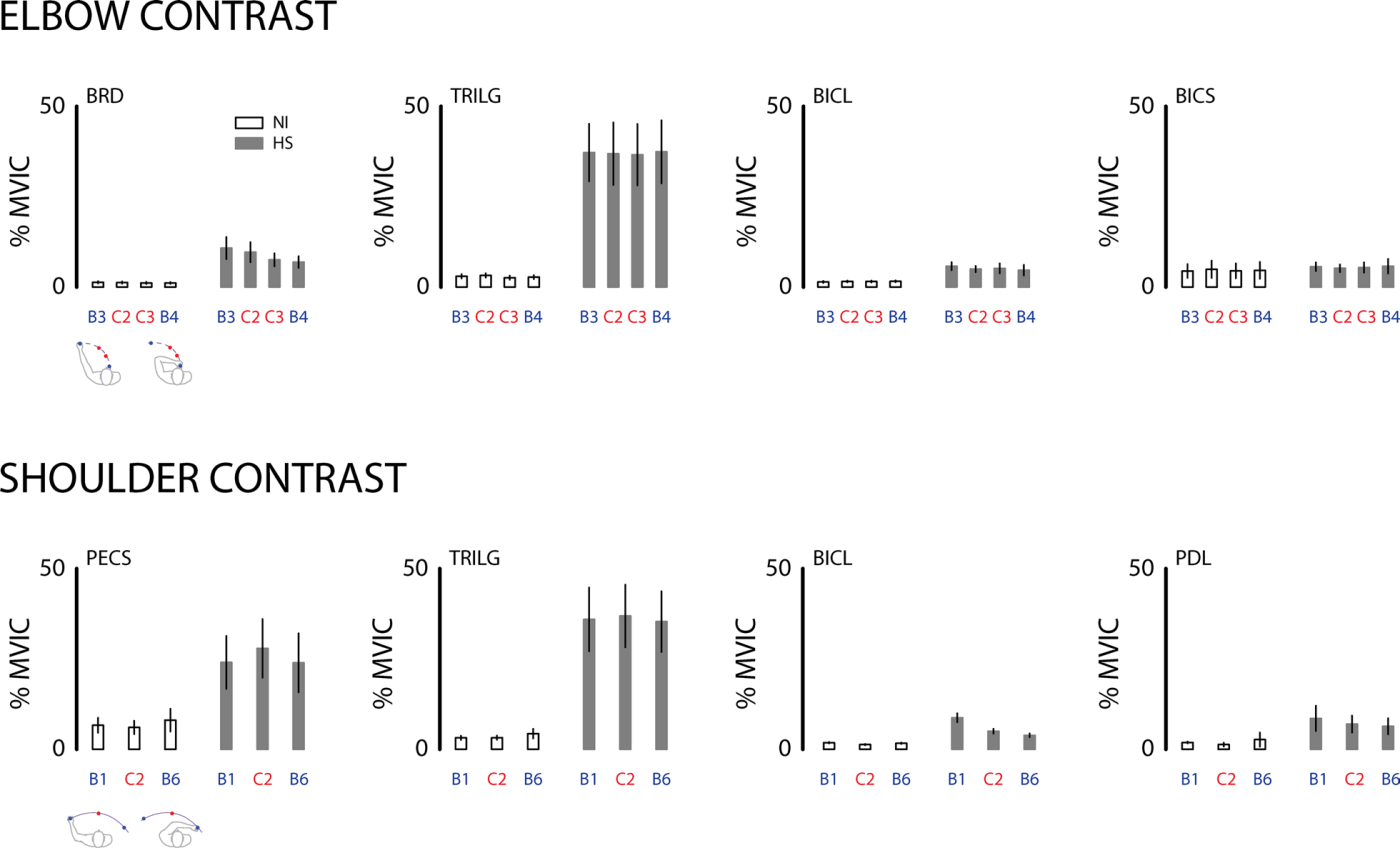
Cohort results: analyses of selected muscle activations (as a percentage of MVIC) at EOT + 2 s in the elbow contrast (top) and shoulder contrast (bottom), as described in the text. **B1, B3, B4, and B6:** selected workspace boundary positions; **C2 and C3:** selected central positions. Icons below the left panels depict limb configurations for the two boundary targets in each contrast. Grey bars: HS; Open bars: NI. Error bars represent ± 1 SEM

These general observations were confirmed with a set of three-way, repeated measures, general linear model ANOVA that examined the extent to which the elbow and shoulder muscle activations varied by subject group and sample time across relevant workspace locations. Results from the elbow analyses (Fig. 7, top) were consistent with our hypothesis that interaction forces induced by passive translation of the hand are partly neuromuscular in origin in that we found a main effect of subject group for BRD (F_(1,115)_ = 8.67; p = 0.010), TRILG (F_(1,115)_ = 12.66; p = 0.003), and BICL (F_(1,115)_ = 6.68; p = 0.020). In each case, the measured muscle activations were a larger percentage of their voluntary maximum capacity throughout the workspace for the HS group as compared to the NI control group. We did not observe a main effect of subject group for BICS or TRILT (F_(1,115)_ < 0.71 and p > 0.411 in both cases). We did however observe an apparent interaction between subject group and target for BRD (F_(3,115)_ = 3.86; p = 0.011) such that for the HS group only, BRD activation was systematically greater when the elbow was extended and that muscle was stretched, vs. when the elbow was flexed and the muscle was shortened. We observed no other two-way interactions for any of the recorded elbow muscles. We also found no main effect related to sample time (F_(1,115)_ < 0.68; p > 0.412 in all cases), or any interaction between sample time and the other two fixed factors (F_(1,115)_ < 0.93; p > 0.336 in all cases), reflecting the fact that the patterns of abnormal EMG seen at EOT + 2 s were also seen at EOT + 20 s.

For the shoulder analyses (Fig. 7, bottom), we found no main effect of sample time (F_(1,82)_ < 0.97; p > 0.328 in all cases), and no interaction between sample time and the other two fixed factors (F_(1,82)_ < 0.93; p > 0.338 in all cases). We found a main effect of subject group for PECS (F_(1,82)_ = 4.70; p = 0.046), TRILG (F_(1,82)_ = 11.12; p = 0.004), and BICL (F_(1,82)_ = 10.98; p = 0.004) in that measured muscle activations were a greater percentage of MVIC throughout the workspace for the HS group as compared to the NI control group. We did not observe a main effect of subject group for PDL or ADL (F_(1,82)_ < 3.01 and p > 0.102 in both cases). We did however observe an interaction between subject group and target for BICL (F_(2,82)_ = 24.24; p < 0.0005) such that for the HS group only, BICL activation was greater when the shoulder was extended and that muscle was stretched, vs. when the shoulder was flexed and the muscle was shortened. We observed no other two-way interactions for any of the recorded shoulder muscles.

**Fig. 8:**
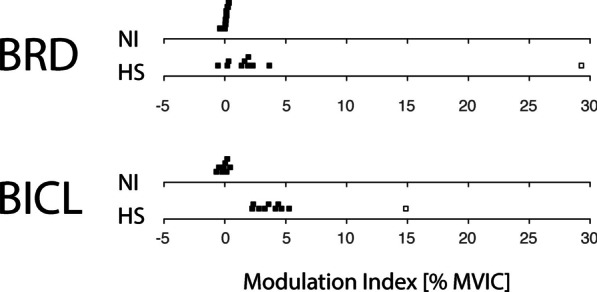
Cohort results: EMG Modulation Index [i.e., the change in normalized EMG values measured across the workspace for the elbow contrast (BRD) and the shoulder contrast (BICL) as described in the text]. Each dot represents the modulation index value obtained from a single subject in either the group of NI control subjects or the group of HS subjects

We examined further the significant interaction effects by calculating for each subject an *EMG modulation index* as the difference between normalized EOT + 2 s EMG values measured at the target location where the muscles were most flexed vs. where the muscles were most extended (Fig. 8). For BRD (elbow contrast), this meant subtracting normalized EMG values measure at target B4 from those measured at B3. For the two-joint muscle BICL (shoulder contrast), this entailed subtracting values measure at target B6 from those measured at B1. EMG modulation index values were tightly packed around 0% MVIC in the NI control group. By contrast, index values were greater than zero in both the BRD and BICL muscles in the HS group. While a single HS appeared to be an outlier in both cases (BRD: HS09; BICL: HS02) (Fig. 8; open squares), removing these outliers and repeating the ANOVA described above did not impact the pattern of main and interaction effects reported. Thus, we obtained support for the idea that passively stretched muscles held in an elongated vs. shortened state elicit greater involuntary activations after stroke in a subset of tested muscles. Neither modulation index exhibited significant correlation with either FMUE or MAS scores.

## Discussion and conclusions

We used a planar robot to assess the spatial and temporal topography of position-dependent hand forces and electromyographic activations that arise during and after passive movement of the arm after stroke. Survivors of hemiparetic stroke (HS) and neurologically intact (NI) control subjects were instructed to relax as the robot repositioned the hand at several different testing locations within a horizontal planar slice through the hemiparetic arm's passive range of motion. The robot was programmed such that movements to each testing location could occur from at least two different directions and at three different speeds ranging from very slow (6°/s) to fast (90°/s). The robot held the hand at the testing location for a minimum of 20 s after each transition. We recorded hand force and electromyographic activations in selected muscles spanning the shoulder and elbow joints throughout the transition and during the holding period that followed. All of the HS and a subset of the NI subjects returned to the lab on subsequent days to repeat the testing procedures. We found that hand interaction forces were relatively small at all speeds and sample times in NI subjects, whereas they varied systematically by transport speed during and shortly after movement in HS subjects. Observation of velocity-dependence after movement had ceased indicates that hand forces were not solely due to passive tissue viscoelasticity post-stroke, but rather implicated the presence of abnormal stretch reflexes. In the HS group, spastic responses (i.e. velocity-dependent resistance to stretch) diminished within 2 s after the end of transition (EOT). Hand forces changed very little from EOT + 2 s to the end of the sampling period (EOT + 20 s), exhibiting dependence on limb posture but no systematic dependence on movement speed and/or movement direction. Although each HS subject displayed a unique field of hand forces across the arm’s reachable workspace after movement ceased, the magnitude of steady-state hand force was greater near the outer boundaries of the workspace than in the center of the workspace for the HS group but not the NI group. In the HS group, hand forces were stronger on one side of the reachable workspace and pointed towards an equilibrium posture located in the approximate center of the workspace. These results were repeatable across testing days. We consider the steady-state hand forces to reflect abnormal postural biases because they depend on history of stroke and limb configuration but not on the speed nor direction of passive movements bringing the hand to the different spatial sampling locations.

Stroke-related postural bias forces were partly neuromuscular in origin because they were accompanied by persistently elevated EMG activation levels in most muscles spanning the shoulder and elbow joints (Fig. 7). During and shortly after movement, passive translation of the hemiparetic arm led to increased EMG activations during movement consistent with spastic hypertonia (e.g., [41]. In some muscles, elevated muscle activation could remain active long after motion had ceased (cf., Fig. 2A, left), as might be expected due to a decrease in the stretch reflex threshold post-stroke [23, 24]. These neuromuscular responses to passive displacement of the hand were persistent in the sense that the complex spatial patterns of EMG activations observed at EOT + 2 s were also observed at EOT + 20 s. The specific details as to which muscles exhibited increased activations varied from person to person, although some general trends were apparent. Across subjects in the HS group, muscles that exhibited posture dependent activations at EOT + 2 s also did so at EOT + 20 s (BRD, BICL). Muscles that exhibited elevated activation throughout the workspace at EOT + 2 s also did so at EOT + 20 s (TRILG, PECS). Muscles that did not exhibit elevated activations at EOT + 2 s also failed to do so at EOT + 20 s (BICS, TRILT, ADL, PDL). Persistence of upper extremity muscle activity (sustained spontaneous discharge) has also been observed after voluntary isometric contractions in the hemiparetic arm after stroke [3]. By contrast, persistence of muscle activation was never observed in NI control subjects during or after passive movement (Figs. 6B and 7).

### Disentangling velocity- and posture-dependent responses to imposed arm movement—transient and steady state effects

Schmit and Rymer [41] created a muscle activation model to measure the static and dynamic components of the stretch reflex observed during passive extension of the elbow at 10 different velocities by examining different aspects of the muscle torque/angle relationship. They found that four out of six model parameters reflected the static stretch reflex response, and the remaining two parameters reflected the dynamic stretch reflex response. The mechanism of static tonic stretch reflexes presumably involves receptors that are chiefly sensitive to muscle length and not velocity. The secondary muscle spindle afferents maintain an increased firing over baseline for as long as the muscle is held stretched and would be suitable candidates. Muscle spindle primaries are suitable candidates for mediating the dynamic effects. In another prior study, Trumbower and colleagues compared short- and long-latency reflex responses elicited by small (5°) passive ramp-and-hold displacements of the elbow as HS and NI subjects generated small (~ 5 N) forces against stiff and compliant environments rendered by a robotic device [47]. Whereas NI subjects systematically increased the amplitude of long-latency reflexes when interacting with the compliant (less stable) environment relative to the stiff environment, this facilitation was absent in both arms of stroke survivors, consistent with a reduction in the capacity for task-dependent modulation of long-latency reflex activation.

Our findings extend the results of these previous studies in that hand forces in steady state after displacement were almost exclusively position dependent in our cohort of stroke survivors, with no measurable contributions attributable to movement speed, direction, or repetition across days. Though we also observed significant velocity-dependent responses during and shortly after passive translation of the hand, the current study focused on steady-state mechanical and electromyographic responses rather than on transient responses because prior studies of goal-directed reaching found that deficits in the control of limb posture and movement were dissociable after stroke [27, 40]. We therefore sought to characterize the spatial topography of position-dependent hand forces and electromyographic activations that arise even with the arm at rest. We observed no residual velocity-dependence in hand forces measured at time points exceeding 2 s after the end of hand translation. Although the details of the spatial topography of hand forces were subject-dependent in the HS group, we observed systematic variations in that forces were greater along the boundary of the arm's workspace than in the center of the workspace (Fig. 5B). These increased bias forces were neuromuscular in origin—at least in part—because regions of elevated hand forces corresponded with regions of elevated muscle activation in some of the muscles spanning the shoulder and elbow joints (e.g., Fig. 7; BRD and BICL; Fig. 8). Although other muscles also exhibit abnormal activations throughout the workspace (e.g., TRILG, PECS, PDL), the measured bias forces reflect a mechanical contribution from elbow flexors (as in Fig. 5A) consistent with the expression of the classic abnormal flexion synergy [4].

### Spatial topography of postural biases

Others have also studied the spatial topography of EMG activations elicited by passive displacements of the arm post-stroke, albeit with limited spatial resolution. Using “quasi-static” (< 5º/s) passive stretches applied to the elbow starting from full elbow flexion or extension and with the shoulder held at three joint angles, Musampa and colleagues [30] identified the elbow joint angles at which different flexor and extensor muscles would begin to be activated. They found that static stretch reflex thresholds encroached on the active range of motion post-stroke and varied significantly with initial shoulder angles. This effect was not observed in neurologically intact control subjects. The authors attributed these observations to the presence of a tonic stretch reflex response at rest due to elevated excitability of α-motor neurons innervating stretched muscles arising from a loss of descending inhibition and the ensuing imbalance of excitation and inhibition (cf. [21, 32]). The authors concluded that a consequence of the encroachment of static stretch reflex thresholds into the arm's typical range of motion is the existence of so-called spasticity zones—i.e., regions of the arm's workspace in which some flexor or extensor muscles cannot be relaxed [30]. For some muscles, spasticity zones occupied a substantial part of the biomechanically defined range of motion. In some cases, the spasticity zones of antagonistic flexor–extensor muscle groups overlapped, yielding what the authors called “spatial co-activation zones” [30]. Other studies have reported that abnormal muscle coactivations and joint torque-coupling patterns constrain the ability of individuals with stroke to voluntarily generate the full typical range of joint torque combinations ([6], cf. [36]). Abnormal patterns of agonist and antagonist muscle activation during stretch or voluntary movement have been previously described in single- and double-joint muscles around the elbow joint in adults and children with hemiparesis [1, 4, 5, 12, 24, 36].

The results of our present study confirm and extend the findings of those prior studies, demonstrating that position-dependent hand forces and EMGs observed post stroke during the static hold phase in our study are indeed neuromuscular in origin—at least in part. Across our cohort of HS subjects (Fig. 7), we observed all three patterns of stretch reflex threshold variations predicted by Musampa et al. [30]. In some muscles (BICS, TRILT, ADL, PDL) we observed no systematically-elevated EMG activation anywhere in the workspace, consistent with static stretch reflex threshold normally established beyond (i.e., greater than) the muscle’s longest length over the joint’s passive range of motion. In other muscles (TRILG, PECS) we observed substantially-elevated EMG activation throughout the entire workspace, consistent with a static stretch reflex threshold abnormally established much shorter than the muscle’s shortest physiological length. Finally, we observed some muscles (BRD, BICL) that exhibited abnormally elevated responses to passive limb displacement in a way that was sensitive to limb configuration such that activation was greater when the muscle was held at longer lengths than when held at shorter lengths (Fig. 8). These results are consistent with the abnormal setting of static stretch reflex thresholds within the joint's normal range of motion. Important questions for future study are whether the passive bias phenomena we observed can compromise volitional control, and if so, whether it may be possible to mitigate their effects, for example, through training to counter the bias forces using strategic antagonist co-contraction.

### Limitations and future directions

Our findings raise several questions that are ripe for further study. For example, the range of muscle activations available for voluntary control should be greater in regions of lower bias force than in regions where bias forces are greater. If so, then there should be systematic interactions between the bias forces measured with the limb at rest and the ability to perform voluntary actions throughout the arm's workspace. Is it easier to stabilize the limb against environmental perturbation in regions of low bias force rather than in regions where bias forces are greater in magnitude? Might moving into a region of greater bias force be more difficult (and less accurate) than moving into a region of lower bias force? Preliminary data suggest that such interactions may indeed arise after stroke [20, 42]. Indeed, a recent study of transcutaneous auricular vagus nerve stimulation suggests that this form of non-invasive stimulation can reduce undesired antagonist activations that arise during goal directed reaching when stimulation is applied during the planning of arm extensions [2].

The current study also has several limitations. First, the focus of our study was only to develop and test a novel robotic approach for quantifying how displacing the resting hemiparetic arm gives rise to elevated resultant hand forces that vary markedly across the reachable workspace due to abnormal neuromuscular mechanisms. Although we do not foresee an immediate clinical application for this basic science technique, we speculate that more effective and individually-targeted therapeutic interventions will evolve from a better understanding of the specific deficits in the control of limb posture and movement that may arise depending on the locus and extent of each individual’s specific stroke-related injuries. A related limitation is the amount of time required to obtain postural bias maps using the procedure described in the current study. Any future clinical application must be fast as well as accurate. As we have shown however, measured forces and muscle activations achieve steady state 2 s after the end of passive displacement of the hand. We also found very consistent results when we transported the arm at 90°/s. We therefore suggest that useful posture maps could be obtained using brief holding periods between passive displacements performed at that fast speed. Doing so could reduce testing time by at least one order of magnitude. Yet another limitation is that we only assessed postural bias in the horizontal plane with the hand supported against gravity by the robot. Although a full 3D volumetric assessment would be intriguing to examine, obtaining it would undoubtedly take a long time even with abbreviated holding times and fast transport speeds.

Finally, the postural biases identified here during passive transport of the arm may be very different from those that would be mapped if the limb were active under volitional control. One way to determine if this were so would be to require subjects in a future study to generate low levels of cued co-contraction 2 or 3 s after the passively-displaced limb comes to rest at each desired location in the workspace. By co-contraction, we mean the condition where the subject would generate some minimum activation (e.g., 5% MVIC) in opposing flexor/extensor pairs spanning the joints of interest. Although the resulting hand forces and muscle activations would be modulated by factors including the imbalance of weakness across flexor and extensor muscles and deficits in the coordination of the muscle pairs, comparison of the postural bias maps obtained after passive displacement and during active generation of modest coactivations could yield insight into whether volitional control is constrained in a manner consistent with an impact of postural biases.

## Data Availability

The datasets generated and/or analyzed during the current study are not publicly available due to potential HIPPA concerns but are available from the corresponding author on reasonable request.

## Appendix 1

See Table 1

**Table 1:** Subject characteristics

Subject	Sex	Age	Year post stroke	Stroke Type	Paretic Side	MAS	Impairment	FM	Elbow		Shoulder		Handed
									min flex	max ext	min flex	max ext	
HS01	F	54	9	I	L	1.25	Mild	53	60	175	70	160	R
HS02	F	58	25	H	L	2	Severe	19	60	175	70	160	R
HS03	F	63	5.7	I	L	0.5	Mild	40	60	175	70	160	A
HS04	F	53	5.9	H	L	2.5	Moderate	27	60	175	70	160	R
HS05	M	49	6	I	L	2	Severe	23	35	95	80	130	L
HS06	M	59	2.10	I	R	1	Moderate	38	100	40	100	30	R
HS07	M	52	28	H	L	1.75	Severe	24	80	165	70	160	R
HS08	F	53	12	I	L	2.5	Moderate	31	80	165	70	160	R
HS09	M	61	3	I	R	3.5	Moderate	29	100	40	100	30	R
HS10	M	64	8	I	L	2	Mild	43	80	165	70	160	R
NI01	M	43							100	40	100	30	R
NI02	F	52							100	40	100	30	R
NI03	M	49							100	40	100	30	R
NI04	M	45							100	40	100	30	R
NI05	M	63							100	40	100	30	R
NI06	F	60							100	40	100	30	R
NI07	F	58							100	40	100	30	R
NI08	M	58							100	40	100	30	R
NI09	F	55							100	40	100	30	R

*Stroke Type: I* ischemic stroke, *H* hemorrhagic stroke, *MAS* modified Ashworth score, averaged across joints [e.g. if elbow flexion = 1 and elbow extension = 1 + , then MAS = (1 + 1.5)/2], *FM* Fugl Meyer Score, upper extremity portion (max = 66), *EF* elbow flexion, *EE* elbow extension, *SF* shoulder flexion, *SE* shoulder extension, *Handed* handedness, *R* right, *L* left, *A* ambidextrous
